# Analysis and optimization of the adsorption of lead ions on a new material based on silica synthesized from blast furnace slag

**DOI:** 10.55730/1300-0527.3705

**Published:** 2024-11-18

**Authors:** Toufik CHOUCHANE, Ouahida KHIREDDINE, Sabiha CHOUCHANE, Mohamed Tayeb ABEDGHARS, Hazem MERADI

**Affiliations:** 1 Research Center in Industrial Technologies CRTI, Algiers, Algeria; 2 Department of Chemistry, Faculty of Sciences, Badji Mokhtar University, Annaba, Algeria

**Keywords:** Adsorption, lead, kinetics, slag, silica

## Abstract

A novel silica-based material (SBM), synthesized from chemically-, thermally-, and mechanically-treated blast furnace slag (TBFS), was examined for its batch-mode lead adsorption capacity based on various parameters. Physicochemical examinations revealed that the formulation of the new SBM consisted mainly of silica, which represented 81.79% of its total composition. After modification, the measured specific surface area changed significantly, from 275.8 to 480.13 m^2^/g, with a point of zero charge (PZC) of approximately 3.4 on the pH scale. The experiment revealed that the driving factors (contact time, stirring speed, solution pH, temperature, and initial concentration) greatly influenced improvement of the lead adsorption capacity, which reached 164.84 mg/g after 40 min of interaction. The adsorption isotherms demonstrated that the lead adsorption took place on a homogeneous surface and in a single layer, which was confirmed by the correlation coefficient and the ability of the Langmuir model to adsorb. The separation factor (R_L_) and heterogeneity factor (1/n) demonstrated that adsorption was favorable, while the Temkin parameter (b_t_) revealed that removal occurred through physical adsorption. According to the kinetic analysis, this process followed a pseudo-second-order kinetic model and was regulated by both external diffusion and intraparticle diffusion. Thermodynamic parameters demonstrated that lead adsorption was a spontaneous, exothermic, less entropic, and physical process, driven by electrostatic interaction. Activation energy revealed that the lead removal process occurred through physical adsorption. Desorption analysis demonstrated that SBM can be reused up to four consecutive times.

## 1. Introduction

Water contaminated with heavy metals currently represents a major global challenge, causing damage to humans and ecosystems. It is true that toxic metals, including cadmium, mercury, arsenic, lead, chromium, nickel, zinc, and manganese, are continuously released into the environment, causing serious harm to wildlife. and flora. This pollution will have harmful consequences for the economy and the health of populations [1–3]. In order to ensure a healthy environment and protect water, which is a precious natural resource, it is essential to apply rigorous sanctions against all pollution. To achieve this, it is crucial to rely on cutting-edge technological methods such as ion exchange, liquid-liquid extraction, reverse osmosis, ultrafiltration, the extraction of magnetic nanomaterials, and adsorption [4–6]. In recent times, emphasis has been placed on the use of affordable adsorbents such as natural materials and industrial solid waste in this process [7,8]. In this context, this research aimed to eliminate the lead contained in water using adsorption on an innovative silica-based material (SBM), produced from treated blast furnace slag (TBFS) at the steel complex of El-Hadjar in Algeria.

Lead is frequently used in several industrial fields, including mining, coating materials, and the manufacturing of zinc, batteries, metal mixtures, and nonferrous metals. It is recognized as being very harmful to humans and ecosystems. The accumulation of this substance in the organs can lead to very dangerous side effects, such as the dysfunction of renal blood and neurological systems [1]. Due to these harmful effects, the World Health Organization and the International Agency for Research on Cancer have classified lead as an extremely hazardous contaminant [9]. For this purpose, many researchers have carried out work in the field of remediation of lead-containing water by adsorption processes, wherein they used various adsorbents, including TBFS [10], halloysite nanotubes [11], the covalent organic structure modified by amidoxime [12], Na-type zeolite prepared from coal fly ash [13], chicken eggshell powder [14], apatite extracted from mixed fish bones [15], natural zeolite and chamotte clay [16], and dead sea mud [17].

Blast furnace slag (BFS) is a byproduct of the production of cast iron in a steel blast furnace. It is mainly composed of lime (CaO), silicon (SiO_2_), alumina (Al_2_O_3_), magnesium oxide (MgO), and a low level of metal oxides [10,18]. Its quantity varies depending on the amount of cast iron produced, which is a very heavy burden for production plants. In addition, its open storage could lead to contamination of the soil, air, and groundwater. In developing countries, such as Algeria, its use is particularly focused on road embankment and the production of construction materials, which are not economically profitable. Faced with this worrying situation, several scientific research laboratories have started working on slag with the aim of improving its use, increasing its efficiency, and reducing the pressure on the steel industry. Indeed, it has been observed that they have started to associate it with the production of cement, ceramics, agricultural fertilizers, and solutions for wastewater treatment. According to the scientific literature, in the field of wastewater treatment, properly treated BFS has been considered as a substantial adsorbent to remove harmful contaminants dissolved in water, such as lead, nickel, manganese, copper, trivalent lead chrome mixture, copper, zinc, and cadmium mixture, blue methylene, acidic methyl orange, and phosphate [10,18–25]. This development is undoubtedly indispensable, but it is insufficient for the complete use of BFS. Thus, research has been carried out to further improve BFS as an adsorbent. Indeed, TBFS has served as an effective and reliable tool for the synthesis of several adsorbents, including double-layer hydroxide [25], hydroxyapatite-zeolite [26], silica nanoparticles [27], and oxalated BFS [28]. Hence, these factors facilitated the current research on the modification of slag and its integration into water purification processes.

Herein, the TBFS was converted into a new material mainly composed of silica. The conversion was performed after applying various treatment processes, including chemical, thermal, and mechanical treatments. The decision to synthesize SBM was based on the fact that low-quantity siliceous materials have significant specific surface area, good adsorption capacity, and can be reused multiple times in the same process [27]. In addition, these materials have varied adsorption capacity [9,29] and are environmentally friendly [18]. As another advantage, the main constituent of SBM, BFS, is readily available at low cost. It should be noted that SBMs, such as silica microparticles, silica nanoparticles, and silica gel, have demonstrated the ability to adsorb toxic elements [9,29–35].

This paper aimed to prove that BFS can be successfully converted into SBM. Furthermore, to ensure its efficiency and reliability in the processes of removing toxic pollutants dissolved in water, such as lead, by adsorption. According to the literature, BFS has been widely exploited in the adsorption of toxic pollutants [36]. However, there has been little research on silica materials synthesized from BFS in the past five years [36], especially regarding their use in the adsorption process, thus making the adsorbent herein of superior value. The synthesis and application of this adsorbent have the advantage of being both less expensive and equally effective than commercially available adsorbents.

In this research, the BFS was treated and modified according to specific experimental procedures. The TBFS and SBM samples were physiochemically characterized using X-ray fluorescence (XRF), X-ray diffraction (XRD), and energy dispersive spectroscopy (EDS). The specific surface areas were quantified using the Brunauer–Emmett–Teller (BET) model, carrying out nitrogen adsorption and desorption. The adsorption capacity was measured experimentally by taking into account the influence of influencing factors such as the contact time, stirring speed, pH, temperature, and initial concentration. The Freundlich, Langmuir, and Temkin adsorption isotherm models were employed to unveil the interactions between the lead ions and the adsorbent used. Understanding the adsorption kinetics was made possible using pseudo-order and diffusion models. Examination of the thermodynamic parameters, including free enthalpy, enthalpy (ΔH°) and entropy (ΔS°), made it possible to identify the nature of the adsorption processes accomplished. The use of multiple solvents in the adsorption-desorption process allowed examination of the lead ion desorption and SMB reuse.

## 2. Materials and methods

### 2.1. Materials

The slag samples were collected at the blast furnace and raw materials workshop of the hot zone, El-Hadjar steel complex, Annaba, Algeria. The solid samples were in the form of rocks that were dark gray with white spots.

### 2.2. Treatment of the solid

The BFS samples were treated in accordance with the method of the experimental protocol established by recently published research [10,20]. Overall, the treatment process was performed in several stages, comprising preliminary washing with distilled water, drying in the open air, grinding into a fine powder, sieving to separate the particles according to their size, additional washing with distilled water, steaming at 105 °C, and finally packaging in plastic boxes.

As for the transformation of the TBFS into the new SBM, this was inspired by research previously carried out in the same field [27,28,35,37]. The detailed steps of the experimental method of converting the TBFS into the SBM are given below.

TBFS (50 g) with a particle size of 1 mm was added to a 1-L beaker containing a NaOH solution (1 Mol/L). The solution obtained was subjected to continuous stirring (200 rpm) for one h. After 4 h at rest, the treated solid was recovered via filtration, and then washed twice with demineralized water. The filtrate was steamed at 105 °C for 8 h before being incorporated into a 1-L solution containing phosphoric acid (1.5 Mol/L) with a pH of 2.5. The new mixture was stirred slowly for 2 h at 50 rpm, allowed to rest for 30 min, and then the solid material was recovered by centrifugation. The material recovered then underwent a succession of treatments, namely washing with demineralized water, steaming at 105 °C for 4 h, and heating in a muffle furnace at 600 °C for 5 h. As soon as this phase was completed, the solid obtained was soaked in demineralized water for 2 h, and then recovered by filtration. Before being preserved, the final product was subjected to several treatment processes, including steaming at 105 °C for 8 h, grinding into a fine powder, and adequate sieving. The chemical reagents used in these tests were of high analytical quality, with a purity of 99% (Merck Serono SAS, Lyon, France).

### 2.3. Specific surface area determination

The specific surface areas of the samples of the solids examined, namely the TBFS and SBM, were determined from the quantity of nitrogen adsorbed as a function of its pressure [5]. This process was carried out at the boiling temperature of liquid nitrogen (−196 °C) and under normal atmospheric pressure (760 mmHg) [18,38]. The experimental data of N_2_ gas desorption at 77 K were evaluated with the BET model [21,41].

### 2.4. Adsorption process

Batch mode experiments were performed to examine the adsorption of lead by the SBM in solution. The experimental method was based on adding a certain quantity of SBM to a solution comprising lead. For this, lead nitrate in the form (Pb(NO_3_)_2_, 6H_2_O) was used to prepare the solutions to be handled. Parameters such as the contact time (tc), stirring speed of the medium (Vst), pH of the solution, temperature of the solution (T), and the initial solution concentration (C_0_) were adjusted to optimize the adsorption process. The mass (ms) and particle size of the SBM (Øs) were considered constant from the start, at 1 g and 100 μm, respectively. All the solutions analyzed in the experiment were prepared in 1-L beakers. The protocol was performed by referring to the experimental procedure mentioned in the literature [18]. Below are the specifications of the experiment:

- Contact time (tc) effects: t_c_ = 0–180 min; C_0_ = 30 mg/L; V_st_ = 150 rpm; pH = 5.4; T = 20 °C.
- Action of the agitation speed (V_st_): t_c_ = 70 min; C_0_ = 30 mg/L; V_ag_ = 50, 100, 150, 200, and 250 rpm; pH = 5.4; T = 20 °C; Øs = 100 μm; ms = 1 g.
- Action of the pH: t_c_ = 70 min; C_0_ = 30 mg/L; V_st_ = 150 rpm; pH = 3.6, 4.3, 4.8, 5.4, 6, and 6.5; T = 25 °C; Øs = 150 μm; ms = 1 g.
- Action of the temperature (T): t_c_ = 70 min; C_0_ = 30 mg/L; V_st_ = 300 rpm; pH = 6; T = 20, 40, 50, and 60 °C; Ø = 100 μm; ms = 1 g.
- Action of the initial concentration (C_0_) and evaluation of the maximum adsorbed quantity (qe): t_c_ = 70 min; C_0_ = 30–300 mg/L; V_st_ = 150 rpm; pH = 6; Øs = 100 μm, T = 20 °C; ms = 1g.

Analysis of the lead ions was carried out using a PerkinElmer 3110 atomic absorption spectrometry (PerkinElmer France SAS, Villebon-sur-Yvette, France). The pH of the solution was measured using an Ericsson pH meter (Ericsson France SAS, Massy, France). The adsorbent heating process was carried out in a Nabertherm HT16/17 muffle furnace (Nabertherm GmbH, Lilienthal, Germany). The solution was stirred using a SE-100 model mechanical rod stirrer. The chemical reagents used in the various experiments, such as lead, nickel, and cadmium nitrates, were of 99% purity and analytical grade (Merck Serono SAS).

The two parameters controlling the adsorption of lead on SBM,


(1) $${\text{Q}}_{\text{e}}=\frac{{\text{C}}_{0}-{\text{C}}_{\text{t}}}{{\text{m}}_{\text{s}}}\times \text{V}$$

(2) $$\text{R}=\frac{{\text{C}}_{0}-{\text{C}}_{\text{e}}}{{\text{C}}_{0}}\times 100$$

Here, C_0_ is the initial solution concentration, (mg/L), C_t_ is the solution concentration after a time t (mg/L); C_e_ is the concentration at equilibrium (mg/L), V is the volume of the solution (L) and ms is the adsorbent mass (g).

### 2.5. Point of zero charge (PZC, pH_pzc_)

The PZC parameter is essential to establish the surface charge because it allows the surface charge to be deduced. More precisely, the charge is zero when the pH is equal to pH_pzc_, positive when the pH is lower than pH_pzc_, and negative when the pH is greater than pH_pzc_ [2]. PZC evaluation was carried out by incorporating 0.1 g of silica gel samples into a solution containing potassium nitrate (0.1 M). Different pH were used in this experiment, ranging from 2 up to 12, increasing by 2 units each time. The pH adjustment was carried out by adding a few drops of sulfuric acid (H_2_SO_4_). Under the influence of a temperature of 25 °C and a stirring speed of 200 rpm the mixtures remained for 24 h. The PZC was obtained at the intersection of the ΔpH (pH_f_ – pH_i_) versus initial pH (pHi) plot with the X axis [5,10].

### 2.6. Isothermal adsorption

Specific adsorption isotherm models were used to designate maximum adsorption quantities and to better discern the interdependencies that arise during this process. In order to achieve the study objective, the models of Freundlich, Langmuir, Temkin, and Dubinin-Radushkevich were used.

The Freundlich model was employed to symbolize the adsorption process on heterogeneous surfaces in a reversible system [18]. Its linear form can be represented by Eq. (3):


(3) $$logq_{e}=logk_{1}+\frac{1}{n}logC_{e}$$

Here, qe is the experimental maximum quantity (mg/g), C_e_ is the equilibrium solution concentration (mg/L), k_1_ is the Freundlich constant (mg/g)(mL/mg)^1/n^, and 1/n is the heterogeneity factor.

The Langmuir model is used to model the adsorption process on a monolayer surface, presenting an energetic homogeneity, where each adsorption site can accommodate only one molecule. The linear equation of the Langmuir model is presented in Eq. (4) [10]. The separation factor (R_L_) parameter of the Langmuir model can be determined from Eq. (5) [19]:


(4) $$\frac{C_{e}}{q_{e}}=\frac{1}{q_{max}}C_{e}+\frac{1}{q_{max}k_{2}}$$

(5) $$R_{L}=\frac{1}{1+C_{0}b}$$

Here, q_max_ is the theoretical maximum quantity (mg/g), qe is the experimental maximum quantity (mg/g), C_0_ is the concentration of the initial solution (mg/L), C_e_ is the equilibrium solution concentration (mg/L), and k_2_ is the Langmuir constant (L/mg).

The Temkin isotherm approach is based on the assumption that the heat of adsorption of each molecule in a layer decreases linearly with the degree of coverage, due to interactions between the adsorbed molecules and the adsorbent, and that the adsorption exhibits a homogeneous distribution of binding energies up to a maximum binding energy. Its linear equation is represented by Eq. (6) [7]:


(6) $$qe=B_{T}lnA_{T}+B_{T}lnCe$$

Here, ${\text{B}}_{\text{T}}=\frac{\text{RT}}{{\text{b}}_{\text{T}}}$, A_T_ is the equilibrium binding constant (L/g), R is the universal gas constant (8.314 J/mol/K), T is the temperature at 298K, and b_T_ is the constant related to heat of sorption (kJ kmol^−1^).

The Dubinin-Radushkevich model is commonly used to describe how Gaussian energy distribution is adsorbed on heterogeneous surfaces. His area of expertise is adsorption on a multilayer surface based on the pore-filling mechanism. Its linear equation is represented by Eq. (7) [39]:


(7) $$lnqe=lnq_{DR}-K_{DR}{ɛ}^{2}$$

Here, *k_DR_* is the constant related to the mean free energy of adsorption (mol^2^/kJ^2^), *q_DR_* is the maximum adsorbed quantity (mg/g), and $ɛ=\text{RTln}\left(1+\frac{1}{{\text{C}}_{\text{e}}}\right)$ is the Polanyi potential.

### 2.7. Kinetic adsorption experiment

The kinetics of the lead sorption in solution on the SMB prepared from TBFS were addressed by applying renowned models in the field of solid-liquid adsorption, such as pseudo-first order (PFO), pseudo-second order (PSO), intraparticle diffusion (IPD), and external diffusion (ED). Eqs. (8)–(11), respectively, describe the linear forms of the PFO, PSO, IPD, and ED models [20]:


(8) $$\text{ln}(q_{e}-q)=-k_{3}t+lnq_{e}$$

(9) $$\frac{t}{q}=\frac{1}{k_{4}q_{e}^{2}}+\frac{t}{q_{e}}$$

(10) $$q=k_{5}\sqrt{t}+C_{int}$$

(11) $$logC_{e}=k_{6}t+C_{ext}$$

Here, q and qe are the quantity adsorbed at time t and at equilibrium (mg/g), t is the time of adsorption process, k_3_ is the PFO constant (min^−1^), k_4_ is the PSO constant (g/mg min), k_5_ is the IPD constant (mg/m. min^½^), C_int_ is the intercept, k_6_ is the ED constant (min^−1^), and C_ext_ is the intercept.

### 2.8. Thermodynamic study

The thermodynamic study was carried out under optimum conditions (t_c_ = 70 min; C_0_ = 240 mg/L; V_st_ = 150 rpm; pH = 6; Øs = 10 μm, ms = 1 g) at different temperatures, namely 293, 313, 333, and 363 K. Evaluation of the Gibbs energy (ΔG°), ΔH°, and ΔS° were calculated using Eqs. (12)–(14) [23,37]. The activation energy (Ea) was defined using Eq. (15) [5]:


(12) $$\Delta {\text{G}}^{0}=-{\text{RTlnK}}_{\text{d}}$$

(13) $${\text{Lnk}}_{\text{d}}=\frac{\Delta {\text{H}}^{0}}{\text{R}}\times \frac{1}{\text{T}}+\frac{\Delta {\text{S}}^{0}}{\text{R}}$$

(14) $${\text{k}}_{\text{d}}=\frac{{\text{C}}_{\text{i}}-{\text{C}}_{\text{e}}}{{\text{C}}_{\text{e}}}\times \frac{\text{V}}{\text{M}}=\frac{{\text{q}}_{\text{e}}}{{\text{C}}_{\text{e}}}$$

(15) $${\text{Lnk}}_{4}=\text{lnA}-\frac{{\text{E}}_{\text{a}}}{\text{RT}}$$

Here, the ΔG° is in kJ/mol, the ΔS° is in kJ/mol.K, the ΔH° is in kJ/mol, R is the universal gas constant (8.314 J/mol.K), K_d_ is the distribution coefficient (L/g), k_4_ is the PSO constant at the temperatures examined (g/mg min), T is the absolute temperature (K), and A is the frequency factor.

### 2.9. Desorption process

The analyses of the desorption process as well as the reuse and valorization of the SBM were carried out with various solvents, including hydrochloric acid (HCl), H_2_SO_4_, and HNO_3_, at a concentration of 0.1 Mol/L. This approach was carried out following the experimental methodology recommended by Chouchane et al. [20]. This process was accomplished by stirring (200 rpm) over a period of 70 min. The saturated SBM was collected by filtration and steamed at 105 °C before its exploitation in the desorption process. The mass used was 0.1 g, and the solution volume was 100 mL. The efficiency of the desorption process was calculated using Eq. (16) below:


(16) $$Desorption\;rate=\frac{q_{des}}{q_{ads}}\times 100$$

Here, (q_ads_) is the adsorbed quantity at equilibrium (mg/g) and (q_des_) is the desorbed quantity at equilibrium (mg/g).

## 3. Results and discussion

### 3.1. Characterization of the adsorbent

**Figure 1:**
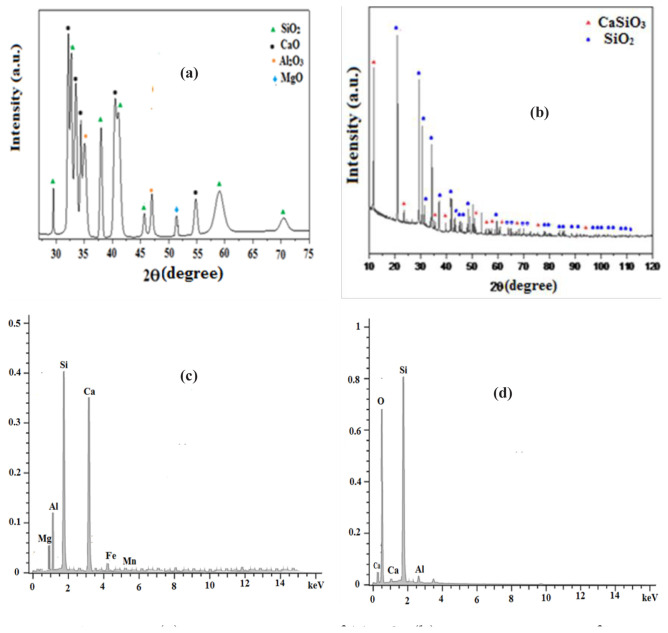
(a) XRD spectrum of TBFS, (b) XRD spectrum of TBFS, (c) EDX spectrum of SBM, and (d) EDX spectrum of SBM.

The solid samples examined, namely the TBFS and SBM, were identified by XRF (Siemens SRS 3000; Siemens Energy SAS, Saint-Denis, France), XRD (Rigaku Ultima IV, Rigaku Europe SE, Neu-Isenburg, Germany), and EDS (Zeiss EVO MA25; Carl Zeiss AG, Oberkochen, Baden-Württemberg, Germany). Their mass percentages are shown in Table 1. The characterization by XRD is illustrated in Figures 1a and 1b, and the spectra performed by EDS are shown in Figures 1c and 1d.

The physicochemical investigations accomplished by XRF of the TBFS were similar to the results mentioned in new publications [19]. The TBFS was made up of 35.21% CaO, 40.85% SiO_2_, 11.38% Al_2_O_3_, 5.03% MgO, and a small proportion of metal oxides (1.04% manganese(II) oxide (MnO), 0.2% potassium oxide (K_2_O), and 0.99% sodium oxide (Na_2_O); Table 1). Figure 1a shows that the XRD analysis results were consistent with those provided by the XRF analysis, revealing significant peaks of CaO and SiO_2_, as well as, to a lesser extent, Al_2_O_3_ and MgO. The EDS analyses, presented in Figure 1c, showed high concentrations of SiO_2_, calcium, oxygen, and to a lesser extent aluminum and magnesium, confirming that the TBFS was mainly composed of CaO, SiO_2_, Al_2_O_3_, and MgO in various proportions.

Table 1 shows that the conversion of the TBFS to SBM increased the mass percentage of SiO_2_ from 40.1 to 81.79%, accompanied by a reduction of CaO, Al_2_O_3_, and MgO, respectively, from 35.45 to 6.56%, 12.96 to 2.11%, and 5.12 to 0.86%. From this first phase, it was established that the conversion of the BFS produced a SBM. XRD analysis of the SBM revealed the presence of peaks corresponding to SiO_2_ and wollastonite (CaSiO_3_) (Figure 1b). This identification allowed for validation of the results provided by the XRF, being aware from the beginning that CaSiO_3_ contains SiO_2_, CaO, Al_2_O_3_ and traces of metal oxides [40]. It should be clarified that the absence of minor metal ions in the spectrum was explained by their dissemination within the SBM structure [37]. Figure 1d shows that the SBM was mainly composed of SiO_2_, oxygen and a small amount of calcium and aluminum. The significant presence of SiO_2_ and oxygen in the EDS spectrum suggests that the SBM was mainly composed of silica. From these multiple investigations, it can be concluded that transformation of the TBFS into the SBM was carried out efficiently.

**Figure 2:**
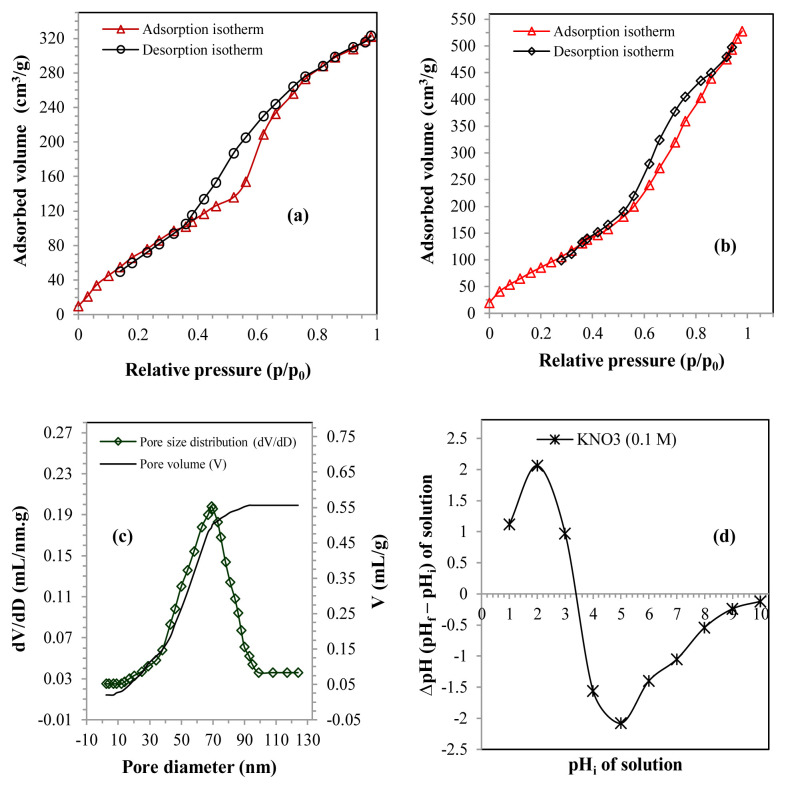
(a) N_2_ adsorption-desorption isotherm of the TBFS, (b) N_2_ adsorption-desorption isotherm of the SBM, (c) SBM pore size and volume curve, and (d) zeta potential as a function of solution pH.

The surface area tests proved that the adsorption and desorption of nitrogen gas corresponded to a type IV isotherm (Figures 2a and 2b), which emphasized the tendency of SBM to have a mesoporous structure with H3 hysteresis loops. This type of isotherm was related to capillary condensation occurring in the mesoporous structure. The specific surface area obtained was 275.8 m^2^/g for the TBFS and 480.13 m^2^/g for he SMB. Figure 2c shows that the pores of SBM displayed an average diameter of 6.92 nm and a volume of 0.554 mL/g, thus confirming the microporous nature of the SBM.

The identification of the zero load point (PZC or pHPZC) for the SBM is presented in Figure 2d, which shows that the intersection of the plot with the X-axis (pHi) was located at 3.4, which lead to the conclusion that this value symbolized the PZC.

### 3.2. Contact time effect

**Figure 3:**
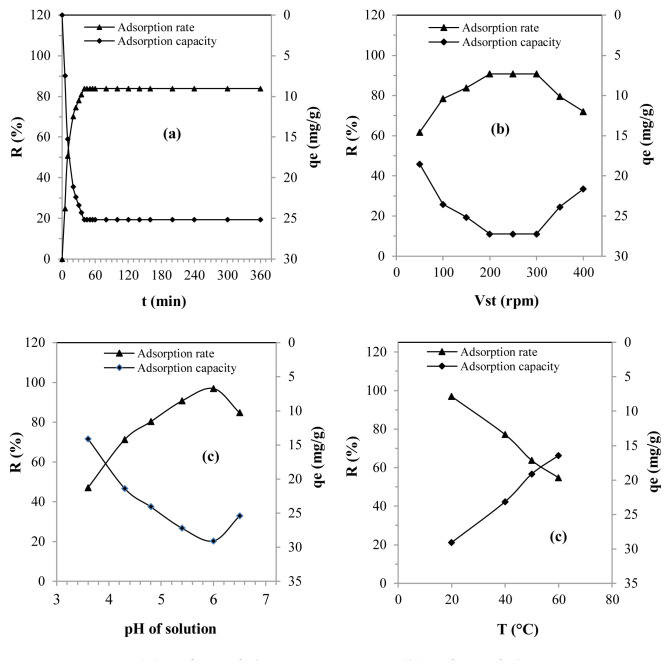
(a) Effect of the contact time, (b) effect of the agitation speed, (c) effect of the initial pH, and (d) effect of the temperature.

The effect of the contact time on adsorption of the lead on the SBM is shown in Figure 3a. According to the experimental tests, the saturation time of the adsorbent surface was achieved after 40 min.

Figure 3a shows that this process went through three stages, namely fast, moderately slow, and stable. In only 20 min (fast step), an increase was observed in the adsorption rate to 70.26% and an adsorbed amount of 21.08 mg/g, most likely due to the abundant presence of active adsorption sites [2].

The reduction in lead adsorption efficiency between 20 and 40 min (medium slow stage) was probably attributable to the gradual reduction of active sites on the surface of the adsorbent [17]. During this stage, the adsorption capacity increased by only 4.08 mg/g, while the yield increased by only 13.6%.

However, during the last stage, the adsorption rate and sorption capacity remained unchanged, despite the extension of the contact time from 40 to 360 min. This can be attributed to the absence of free adsorption sites, resulting from saturation of the adsorbent surface [5].

A literature review revealed that lead removal by adsorption onto the SBM proceeded at a moderately high rate (40 min). Effectively, the lead sorption on various adsorbents, in particular BFS [10], controlled surface multifunctional groups over halloysite nanotube [11], amidoxime-modified covalent organic framework [12], chicken eggshell powder [14], apatite extracted from mixed fish bone [15], dead sea mud [17], castor seed shell waste [41], and low-temperature sulfonated biochar from Macauba’s endocarp [42], was carried out after respective durations of 50, 10, 30, 120, 60, 180, 123, and 120 min.

### 3.3. Effect of the agitation speed

Mixing the aqueous medium is essential in the adsorption removal process, as it greatly promotes the ED of the pollutant from the solution to the adsorbent material [19]. With this in mind, the stirring speeds herein ranged from 50 to 400 rpm with an interval of 50 rpm (Figure 3b).

The experimental tests proved that lead adsorption was more advantageous at 200 rpm. Effectively, it was found that between 50 and 200 rpm, the adsorption efficiency and capacity increased continuously, from 18.53 to 27.22 mg/g and 61.76 to 90.73%, respectively (Figure 2b). It is quite possible that the progressive improvement in the lead sorption efficiency was induced by an increase in the diffusion coefficient, thus leading to increased diffusion of lead ions from the solution toward the adsorbent surface of the SBM [24,43,44].

Figure 3b shows that the lead removal process by adsorption on the SBM stopped working between 200 and 300 rpm, even though the stirring speed increased (27.22 mg/g, 90.93%). It is undeniable that this phenomenon was achieved through the suspension of ED, which triggered the removal of the liquid barrier to generalized diffusion [20]. Based on this, a stirring speed of 300 rpm was chosen as the most suitable.

The experiments carried out further indicated that from 350 rpm, the performance of the process gradually decreased with the intensification of agitation of the medium (Figure 3b). It is highly likely that this was caused by the intense vibrations of the SBM particles, which triggered the partial desorption of the lead [18,20,45].

### 3.4. Effect of the initial pH

The pH of the solution plays a decisive role in the process of sorption of harmful metals, especially lead. Indeed, the pH has a direct hold on the adsorbent surface of the solid and also on the structure of the metal ion in solution. With this in mind, a variety of pHs, ranging from 3.6 to 6.5, were examined (Figure 3c).

A pH below the PZC was not chosen for two reasons. First, the positively charged surface will cause a repulsive effect [18,46]. Second, the presence of several H^+^ protons will decrease the lead adsorption by the competition effect [20,47].

Figure 3b shows that clear improvement in lead removal efficiency was observed when the pH exceeded the pH_PZC_ value. Increases of 14.95 mg/g in terms of the yield and 49.83% in the amount eliminated between a pH of 3.6 and 6 were recorded. It is very likely that the efficiency of the adsorption was attributable to the progressive deprotonation of the solution, which had the consequence of reducing the competition between the H^+^ ions and the Pb^+2^ ions [47]. In addition, the negative charge of the adsorbent surface (SBM) significantly facilitated adsorption through electrostatic interactions [37].

Figure 3c shows that the adsorption rate decreased at a pH of 6.5. The rate and quantity that was eliminated decreased from 93.08% to 83.76% and from 29.08 to 25.43 mg/g, respectively. The decrease in adsorption efficiency was undoubtedly caused by precipitation of the lead in hydroxide form. According to literature data, the formation of lead hydroxide gradually begins to occur at a pH >6 [48,49]. From this, it was deduced that the adsorption of lead on the MSB in the solution was optimal at a pH of 6. Note that similar results were mentioned in the literature [17,41,42,48].

### 3.5. Effect of the temperature

In this section, the impact of temperature on lead adsorption by the SBM was examined using various solutions at varying temperatures, including 20, 40, 50, and 60 °C (Figure 3d).

The experiments clearly proved that the adsorption efficiency of lead decreased as the temperature of the medium increased. Moreover, the two process performance metrics, yield and adsorption capacity, decreased by 28.73% and 8.62 mg/L, respectively, when the temperature increased from 20 to 60 °C (Figure 3d).

From this, it was concluded that the lead elimination by sorption on the SBM was exothermic [5,23,50]. It is likely that the decrease in adsorption efficiency, due to the increase in temperature, was caused by the random movements of lead in the solution and/or by the decrease in the thermal energy of the adsorbate, which weakened the influence of the electrostatic interaction [37,51].

### 3.6. Effects of the initial concentration

**Figure 4:**
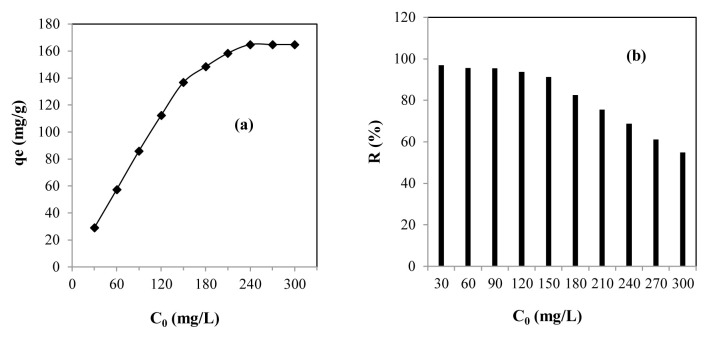
(a) Effect of the initial concentration and (b) lead removal performance by SBM.

Examining the influence of the initial concentration is essential in this process, as it provides the opportunity to evaluate the maximum removal amount from experimentally measured adsorption isotherms. To this end, the impact of the initial concentration on this process was studied for measurements ranging from 30 to 300 mg/L under the operating conditions previously mentioned (Figure 4).

Analysis of the experiments revealed that the adsorption capacity increased with the initial concentration and then remained constant even with the addition of lead ions (Figure 4a). The experimental adsorption capacity increased from 29.08 to 164.84 mg/g for solutions with concentrations ranging from 30 to 240 mg/L. This was possibly due to the formation of strong driving forces that lessened the resistance to mass transfer and, therefore, facilitated the transport of lead ions from solution to the active surface of the SBM [10,52]. Regarding the second step, the interruption of the lead sorption mechanism from C_0_ ≥ 240 mg/L (qe = 164.84 mg/g) was probably caused by the absence of free locations on the active surface of the SBM (saturation) [18–20].

From the results of the experiment, it was also shown that the percentage of lead adsorption on the SBM gradually decreased with the intensification of the initial concentration, from 93.93% to 54.94% (Figure 4b). The gradual weakening of the sorption percentage was surely due to the continuous increase of lead ions in the solution for the same active surface of the SMB. Considering the dependence of the yield on the initial concentration, it was possible to identify this phenomenon. It should be noted that the regression of yield with the intensification of the initial concentration has been discussed in several studies [5,10,18,20,23].

These results showed that the lead adsorption capacity measured when using the SBM (164.84 mg/g) was significantly higher than that measured with the TBFS (50.12 mg/g) [21]. Furthermore, according to the literature data presented in Table 2, SBM demonstrates significant adsorption power compared to various adsorbents such as hierarchical composite adsorbent, chicken eggshell powder doped, chicken eggshell doped, natural zeolite, dead sea mud, sulfonated biochar, tobacco leaves, and Amberlyst 15 resin. These data revealed that the use of slag derivatives (i.e. SBM) as adsorbent is not only more efficient but also constitutes a more advantageous alternative in the treatment of wastewater polluted by lead.

### 3.7. Adsorption isotherms

**Figure 5:**
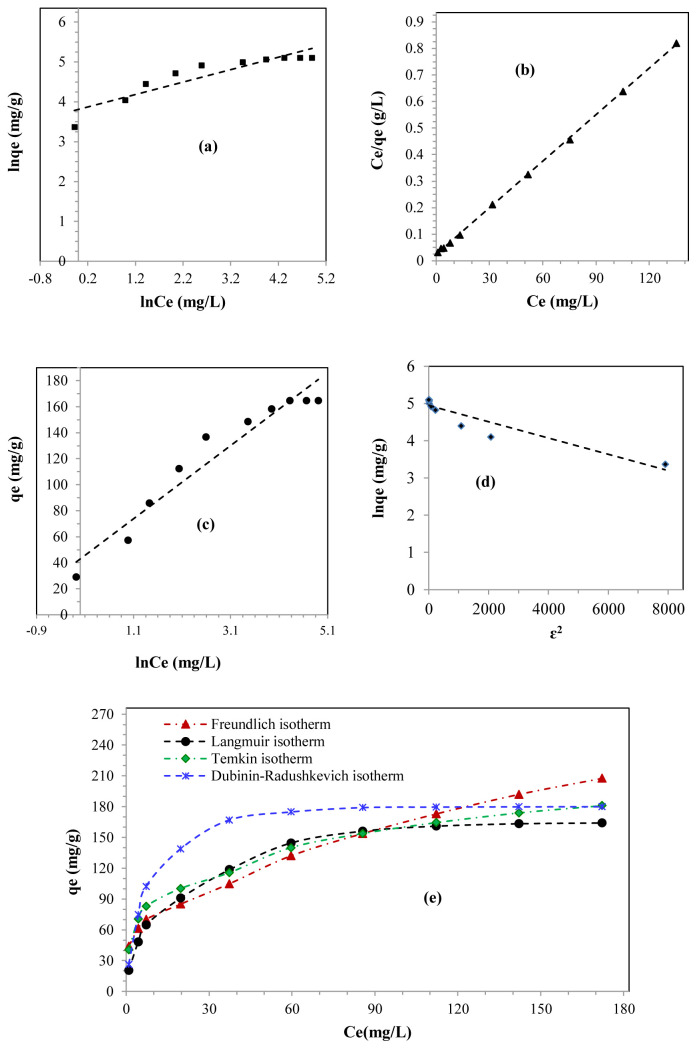
(a) Freundlich model presentation, (b) Langmuir model presentation, (c) Temkin model presentation, (d) Dubinin-Radushkevich model, and (e) curves of the adsorption isotherms for the lead adsorption on SBM.

Figures 5a–5d show the plots of the Freundlich, Langmuir Temkin, and Dubinin-Radushkevich models, respectively. Figure 5e illustrates their adsorption isotherms. The parameters of the models are given in Table 3.

According to the values of the correlation coefficient (R^2^) and the maximum adsorption capacity (q_max_) (Table 3), the Langmuir model was the most suitable for describing the experimental points [10,14,22,53]. Indeed, the Langmuir’s model had a higher correlation coefficient (R^2^ = 0.99) than those of Freundlich (R^2^ = 0.84), Temkin (R^2^ = 0.95), and Dubinin-Radushkevich (R^2^ = 0.87). Furthermore, the value of the theoretical adsorption capacity estimated according to the Langmuir model (q_max_ = 163.94 mg/g) was practically identical to the adsorption capacity measured experimentally (q_exp_ = 164.84 mg/g).

From this, it appears that the Langmuir model was the most relevant for the adsorption of lead on the SBM, followed in order by the Temkin model, then the Dubinin-Radushkevich model, and finally, the Freundlich model (Table 3).

According to Figure 5e, the Langmuir isotherm curve had a shape identical to that of the experimental adsorption isotherm represented in Figure 4a. Furthermore, it was similar to the type 1 isotherm, which confirmed that the adsorption of lead on the SBM was carried out on a homogeneous monolayer surface; that is to say, it obeyed the Langmuir model [29,34,37,54]. The agreement between the experimental data (Figure 4a) and the Langmuir model isotherm (Figure 5a) demonstrated that lead removal on the SBM was accomplished by physical interaction [55,56].

The favorable nature of the lead adsorption process on SBM can be confirmed by the value of the heterogeneity factor (1/n). The adsorption is described as linear when 1/n = 1, it is considered chemical when 1/n > 1, and it is physical and favorable when 0 < 1/n < 1 [27,57]. In the present study, the calculated heterogeneity value was between 0 and 1 (1/n = 0.311), thus indicating that the adsorption was favorable due to the standard distribution of active adsorption sites [10,19,58]. Moreover, it was carried out by physical adsorption [55,57,59]. The separation factor (R_L_) can also be used to determine the quality of the lead adsorption process on SBM. The adsorption is favorable if the R_L_ is between 0 and 1 (0 < R_L_ < 1), unfavorable if it is greater than 1 (R_L_ > 1), linear if it is equal to 1 (R_L_ = 1), and irreversible if it is equal to 0 (R_L_ = 0) [27,57,58]. Herein, the value of the R_L_ was between 0 and 1, which suggests that the adsorption proceeded in a favorable manner (Table 3) [18–21,53,58].

Table 3 shows that the b_T_ value was less than 8 kJ/mol, which also confirmed that the removal of lead by the SBM was achieved by physical adsorption under the effect of electrostatic interactions [24,37,54]. The average energy E, calculated according to the Dubinin-Radushkevich isotherm model ( $E=\frac{1}{\sqrt{2\times k_{DR}}}$), was approximately 7.41 kJ/mol, which also indicated that the removal of lead by the SBM was achieved by physical adsorption, since it was less than 8 kJ/mol [38,50].

From this, it can be deduced that lead ions adsorb on a homogeneous monolayer surface under the effect of weak physical interactions, notably those of van der Waals, which indicates that the process is associated with relatively low adsorption energies [55].

### 3.8. Kinetics of adsorption

**Figure 6:**
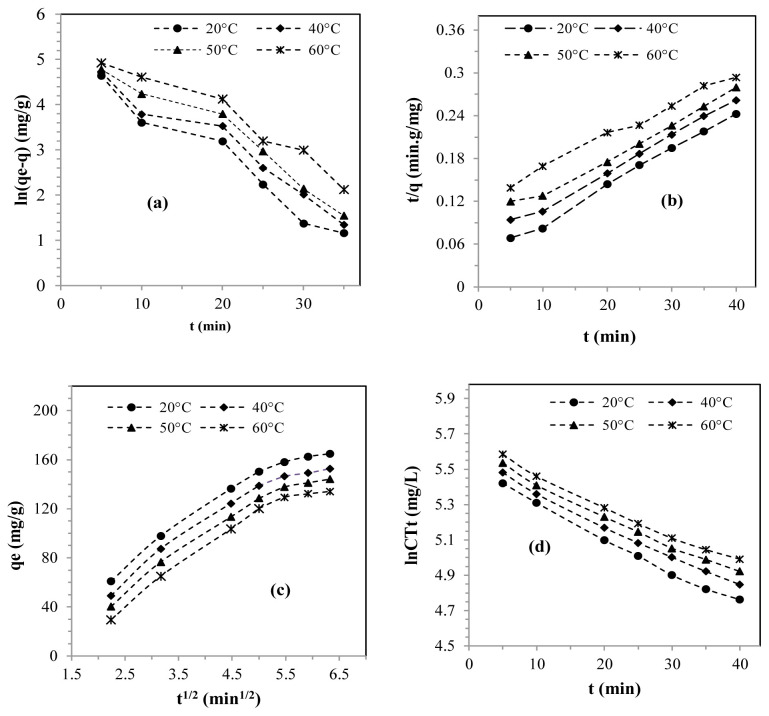
(a) PFO model, (b) PSO model, (c) IPD, and (d) ED.

The kinetics of lead sorption in solution on the SMB was addressed by applying renowned models in the field of solid-liquid adsorption, such as PFO, PSO, IPD, and ED. The graphs showing Eqs. (8)–(11) are illustrated in Figures 6a–6d, respectively. The values of the parameters resulting from Eqs. (8) and (9) are presented in Table 4, while those from Eqs. (10) and (11) are displayed in Table 5.

Table 4 shows that the correlation coefficients of the PSO model (R^2^ ≥ 0.99) were higher than those of the PFO model (R^2^ ≤ 0.95) for the different temperatures studied. Furthermore, the adsorption capacity predictions according to the PSO model were almost equivalent to those provided by the experimental tests (Table 4). Based on this, it was concluded that the lead adsorption process on the SMB followed PSO kinetics [18,46,51,58,60]. The literature also comprises reports of similar cases that followed PSO kinetics for the adsorption of lead in solution by various materials, particularly BFS [10], fine Na-type zeolite [13], sulfonated biochar [42], tobacco leaves [43], silica agar [47], porous cellulose [48], and Amberlyst 15 resin [50].

Figure 6c shows that the curves derived from Eqs. (10) displayed a multilinear shape and did not cross the origin, suggesting that they did not meet the criteria defined by Weber and Morris, as cited in [61]. This suggested that there was more than one mechanism governing the adsorption, with each straight segment representing a specific mechanism [51,58,60]. The first segment describes how lead ions move from the solution to the boundary layer through an ED process, while the second segment highlights the gradual transfer of lead ions from the SBM adsorbent surface to the internal pores through IPD. It is worth emphasizing that the process reaches a steady state and stops when the active adsorption sites are completely filled.

Considering the values of the rate constant (K_5_) and the intercept (C_int_) (Table 5), it can be concluded that the adsorption of lead on the SBM occurred more rapidly during the first stage. Indeed, the values of k_5_ from the first stage were much higher than those obtained in the second stage, while the values of the intercept (C_int_) were the opposite. The adsorption efficiency observed during the first phase could be explained by more efficient mass transfer in the diffusion boundary layer at the start of the process. From these results, it was seen that the adsorption mechanism did not depend only on IPD [33,58,62,63].

Figure 6d shows the curves derived from Eqs. (11), which were in the form of straight lines, with correlation coefficients greater than 0.98 (Table 5). These data revealed that the removal of lead by adsorption on the SBM was also controlled by ED [20,37]. This reaffirmed the hypothesis explained previously. Following these findings, it was established that the adsorption of lead ions on the SBM was initially regulated by ED, followed by an IPD process [58].

### 3.9. Thermodynamic study

**Figure 7:**
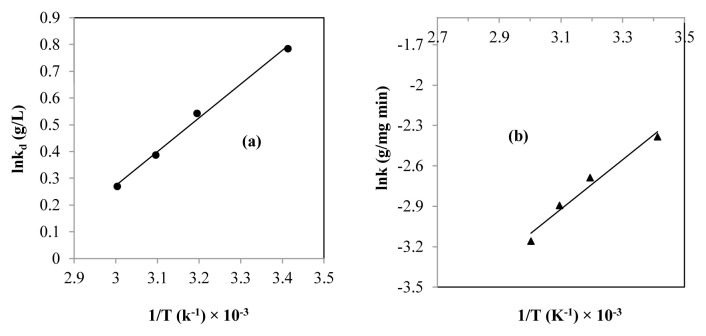
(a) Van’t Hoff plot for lead adsorption and (b) Arrhenius equation presentation.

For this step, a thermodynamic study was performed in order to discern the nature of the lead elimination process and the interaction mechanisms regulating this process. This mainly focused on the evaluation of the ΔG°, ΔH°, ΔS°, and Ea [23,37]. The graphic representations of Van’t Hoff and Arrhenius are shown in Figures 7a and 7b. The values of the ΔG°, ΔH°, ΔS°, Ea, and k_d_ are given in Table 6.

**Figure 8:**
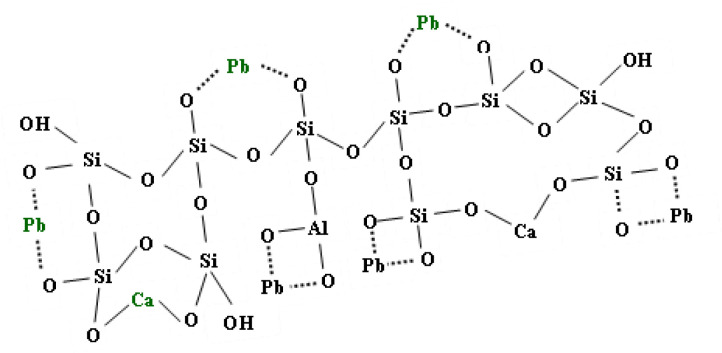
Lead fixation mechanism on the adsorbent surface of the SBM.

According to Figure 8a and Table 6, there was good consistency between the Van’t Hoff model and the experimental results (R^2^ = 0.99). This highlights that the elimination of lead on the SBM was favorable. Due to the decrease in the K_d_ with increasing temperature, the lead adsorption was more favorable in less heated solutions [5,64].

The ΔG° results revealed that the adsorption of lead on the SBM was a spontaneous phenomenon with a negative standard free energy change (Table 6) [37,46,50]. The observation of a decrease in ΔG° as a function of the increase in the temperature of the solution indicates that the mass transfer varied inversely with the temperature of the medium [18,23]. This was in agreement with the observations made during the study on the influence of temperature, wherein the increase in temperature led to a reduction in the adsorption process due to a decrease in the driving force. From the ΔG° values (−18.79 ≤ ΔG°≤ −19.91 kJ/mol), it was established that the removal of lead on the SBM was achieved by physical adsorption under the influence of physical interactions [5,46,58]. It was clearly demonstrated that the adsorption of lead is exothermic, as evidenced by the negative value of the ΔH° (Table 6) [23,46,50]. Still relying on the ΔH° value (≤40 kJ/mol), it was established that lead was eliminated in the solution on the SBM by physical adsorption [18,21,39]. Negative ΔS° analysis revealed the weakening of randomness at the interface of the SBM (adsorbent) and lead ions in the solution (adsorbate) (Table 6) [19,23]. The negative value of ΔS° also demonstrated that the distribution order of metal ions on the SBM progressed compared to that of the solution [65]. According to this hypothesis, the results of the analysis of adsorption isotherms were reaffirmed, showing that there was a regular organization of lead ions on the active adsorption sites, as revealed by the Langmuir model. It is worth mentioning that the cationic interaction between the adsorbent and the adsorbate likely played a role in reducing random motions, thus allowing a better distribution of lead ions on the active surface of the SMB [23,37,66,67].

Considering the Ea value (<40 kJ/mol) (Table 5), it was confirmed that the elimination of lead in solution by the SBM was by physical adsorption [5,50,68].

From these data, it was concluded that lead adsorption on the SMB occurred spontaneously, exothermically, and with low entropic properties. Furthermore, removal occurred by physical adsorption under the effect of electrostatic interactions.

Since the fixation of lead on the adsorbent surface of the SBM occurred under optimal conditions by physical adsorption, influenced by electrostatic interactions, it was assumed that its sorption mechanism was identical to that shown in Figure 8.

### 3.10. Desorption process

**Figure 9:**
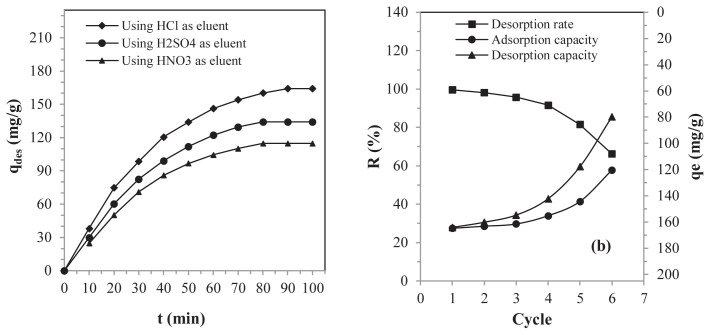
(a) Desorption kinetics and (b) adsorption-desorption cycles.

To ensure the reuse of SBM over multiple cycles, it is essential to remove lead ions from its saturated adsorbent surface. This desorption process also helps to prevent the creation of contaminated solid waste, which could cause another form of pollution. In this framework, the desorption of lead present in the saturated SBM was performed, and the efficiency of this process was calculated using Eq. (15). The kinetics and cycles of the adsorption and desorption of the lead are illustrated in Figure 9.

The experiments carried out revealed that the desorption of lead from saturated SBM was more efficient by involving HCl as the eluent (Figure 9a). The ability of HCl to efficiently desorb lead ions on the SBM adsorbent surface arises from its pronounced polarity and decreased viscosity [23,69]. Figure 9b shows that the desorption of the lead on the adsorbent surface of the SBM was very efficient during the first four cycles, and then its efficiency started to decrease gradually. The electrostatic repulsion produced between the lead ions and the active surface of the positively charged SBM was responsible for the efficiency observed during the first four cycles [23]. The weakening of the desorption process observed from the fourth cycle was surely caused by the depletion of the adsorbent and the loss of material [5]. It is important to note that the desorption process was carried out in a highly acidic environment, i.e. at a pH below the PZC, where the adsorbent surface would be positively charged. In conclusion, the results highlighted that the SBM has considerable potential as an adsorbent and can be efficiently reused over four successive cycles.

## 4. Conclusion

The objective of this study was to investigate the ability of a SBM prepared from TBFS to adsorb lead in batch mode. It was demonstrated by careful examinations that the SBM was composed of 81.79% SiO_2_, with a PZC of 3.4 on the pH scale and a specific surface area of 480.13 m^2^/g. The experiments showed that the adsorption capacity of lead on the SMB reached its highest level (164.84 mg/g) after 40 min in an optimal medium, characterized by a speed of 250 rpm, a pH of 6, a temperature of 20 °C, a mass of 1 g, and a concentration of 240 mg/L. Modeling of the experimental data revealed that the Langmuir isotherm model (R^2^ = 0.99; q_max_ = 163.93 mg/g) was the most appropriate to interpret the sorption of lead on the SMB, thus exhibiting monolayer adsorption on homogeneous active sites. The R_L_ and 1/n constants of the Langmuir and Freundlich models demonstrated that the adsorption conditions were favorable. On the other hand, the b_T_ value of the Temkin model highlighted that the adsorption occurred physically. The kinetic study showed, on the one hand, that the adsorption followed PSO kinetics (R^2^ ≥ 99), and, on the other hand, the transport of lead was carried out by ED and IPD. Lead adsorption on the SBM was a spontaneous, exothermic, and less entropic phenomenon, as indicated by the ΔG°, ΔH°, and ΔS° values. The ΔH° and Ea values showed that the lead removal was by physical adsorption. The desorption process indicated that the SBM was suitable for reuse for four successive cycles, using 0.1 Mol/L of HCl as the elution solvent. Hence, it was concluded that SBM can be exploited as a reliable and efficient adsorbent to remove lead ions from wastewater.

## Figures and Tables

**Table 1:** Chemical composition of the TBFS [16] and SBM.

	CaO	Al_2_O_3_	SiO_2_	Fe_2_O_3_	MgO	MnO	K_2_O	Na_2_O	SO_3_	LOI
	Mass %									
**TBFS**	35.45	12.96	40.1	2.11	5.12	1.04	0.2	0.7	0	3.94
**SBM**	6.56	2.11	81.79	0.42	0.86	0.26	0.23	0.44	0.08	7.25

**Table 2:** Adsorption capacity of lead (II) by various adsorbents.

Absorbent	qe (mg/g)	Reference
Hierarchical composite adsorbent	67.20	[1]
Halloysite nanotube	365.02	[11]
Chicken eggshell powder (doped)	25.1	[14]
Chicken eggshell (doped)	42.7	[14]
Natural zeolite	14	[16]
Dead Sea mud	39.84	[17]
Blast Furnace dlag	50.12	[21]
Sulfonated biochar	104.2	[43]
Tobacco leaves	24.7	[44]
Porous cellulose nanofiber	318.47	[49]
Amberlyst 15 resin	116	[51]

**Table 3:** Isotherm parameters for cadmium adsorption.

Models	Parameters	Values
Freundlich	k_1_ (mg/g)(mL/mg)^1/n^	23.10
	1/n	0.311
	R^2^	0.840
Langmuir	q_max_ (mg/g)	164.93
	k_2_ (L/mg)	0,152
	R_L_	0.017–0.21
	R^2^	0.999
Temkin	b_T_ (kJ/mol)	0.108
	A_T_ (L/g)	3.387
	R^2^	0.952
Dubinin-Radushkevich	k_DR_ (mol^2^/kJ^2^)	0.0091
	q_DR_ (mg/g)	179.32
	R^2^	0.876
	E (kJ/mol)	7.41

**Table 4:** Kinetic parameters of the PFO and PSO models.

Models	Parameters	Values			
PFO	T (°C)	20	40	50	60
	qe_exp_ (mg/g)	164.84	152.64	142.92	136.12
	k_3_ (min^−1^)	0.11	0.098	0.093	0.091
	qe_theo_ (mg/g)	180.18	161.25	157.27	154.93
	R^2^	0.951	0.936	0.943	0.95
PSO	T (°C)	20	40	50	60
	qe_exp_ (mg/g)	164.84	152.64	142.92	136.12
	K_4_ (g/mg min)	0.092	0.068	0.055	0.042
	qe_theo_ (mg/g)	166.66	153.84	144.92	138.88
	R^2^	0.995	0.995	0.99	0.994

**Table 5:** Kinetic parameters of IPD and ED models.

Models	Parameters	Values			
IPD	First step				
	T (°C)	20	40	50	60
	C_int_	7.67	16.7	23.12	37.66
	K_5_ (mg/g.min)	10.33	9.45	9.11	8.44
	R^2^	0.991	0.99	0.99	0.99
	Second step				
	T (°C)	20	40	50	60
	C_int_	65.69	72.56	89.1	96.48
	K_5_ (mg/g.min)	2.11	1.57	1.07	0.71
	R^2^	0.952	0.931	0.937	0.896
ED	T (°C)	20	40	50	60
	C_ext_	5.49	5.54	5.59	5.63
	K_6_ (min^−1^)	0.19	0.18	0.15	0.13
	R^2^	0.993	0.994	0.992	0.987

**Table 6:** Thermodynamic parameters of lead adsorption.

T (K)	ΔH° (kJ/mol)	ΔG° (kJ/mol)	ΔS° (j/mol K)	Ea (kJ/mol)	R^2^	K_d_ (L/g)
293	−10.525	−18.790	−29.315	8.310	0.994	2.193
308		−19.341				1.721
318		−19.653				1.472
328		−19.910				1.310
